# Oral Nintedanib for Refractory Generalized Keloids: A Prospective Exploratory Pilot Study

**DOI:** 10.1111/jocd.71107

**Published:** 2026-08-26

**Authors:** Chenmei Liu, Lian Zhang, Dan Sun, Aiqing Lin, Renliang He, Bin Yang

**Affiliations:** ^1^ Department of Dermatology Dermatology Hospital of Southern Medical University Guangzhou Guangdong China; ^2^ The First School of Clinical Medicine Southern Medical University Guangzhou Guangdong China

**Keywords:** disfiguring dermatosis, generalized keloid, Nintedanib, pathological scarring, systemic therapy

## Abstract

**Background:**

Generalized keloids are severe, treatment‐refractory, disfiguring dermatoses that substantially impair physical and psychosocial well‐being and have limited effective systemic treatment options.

**Objective:**

To assess the short‐term safety, tolerability, and exploratory clinical outcomes of oral nintedanib in patients with refractory generalized keloids.

**Methods:**

In this single‐center, open‐label, assessor‐blinded exploratory pilot study, 10 adults with refractory generalized keloids received oral nintedanib 150 mg once daily or 150 mg twice daily for 8 weeks. The primary exploratory endpoint was change in Vancouver scar scale (VSS) score from baseline to Week 8. Secondary endpoints included pruritus and pain visual analogue scale (VAS) scores, patient and observer scar assessment scale (POSAS), dermatology life quality index (DLQI), imaging and histological assessments, and safety outcomes.

**Results:**

All participants completed treatment and follow‐up. Mean VSS scores decreased from 11.2 ± 1.3 to 8.2 ± 1.1 in the low‐dose group and from 10.4 ± 1.8 to 8.2 ± 1.9 in the high‐dose group. Pruritus severity and visible scar vascularity showed numerical improvements over 8 weeks, whereas POSAS and DLQI changed little. Histological assessment suggested reduced cellularity and more organized collagen architecture after treatment. No serious adverse events or treatment discontinuations occurred; the most common treatment‐related adverse events were mild diarrhea and transient transaminase elevation.

**Conclusion:**

In this small, uncontrolled exploratory pilot study, oral nintedanib was associated with early favorable changes in selected clinician‐rated and symptom‐based outcomes, whereas POSAS and DLQI showed limited short‐term change. These findings are preliminary and hypothesis‐generating and warrant confirmation in larger controlled studies with longer treatment and follow‐up.

## 1 Introduction

Keloids are benign fibroproliferative skin disorders characterized by excessive fibroblast activation, abnormal extracellular matrix (ECM) deposition, and dysregulated angiogenesis [1, 2, 3, 4, 5]. Generalized keloids represent a severe clinical subtype, typically defined by involvement of three or more distinct anatomical sites with a total lesion count exceeding ten. This disfiguring form of disease not only causes persistent and distressing symptoms such as pruritus and pain but also imposes a substantial psychological burden because of its impact on appearance, leading to anxiety, low self‐esteem, and reduced quality of life [6, 7, 8]. Owing to their extensive distribution and complex pathophysiology, currently available treatment modalities, including intralesional corticosteroids, surgical excision, radiotherapy, and laser therapy, often provide limited and unsustained benefits and are associated with high recurrence rates [9, 10]. Effective systemic therapies for generalized keloids remain unavailable, representing a major unmet clinical need.

Advances in the understanding of keloid pathogenesis have stimulated interest in targeted molecular therapies. Several agents with antifibrotic activity have shown potential in preclinical keloid models, including Hedgehog pathway inhibitors, c‐MET inhibitors [11, 12], and tyrosine kinase inhibitors (TKIs) such as sorafenib, nintedanib, and sunitinib [13, 14, 15]. Among these TKIs, nintedanib is of particular interest because of its multi‐target inhibitory effects on fibrosis‐related pathways. However, clinical translation of such agents has remained limited. Early clinical studies of Janus kinase (JAK) inhibitors and anti‐angiogenic agents have shown variable responses, highlighting the biological heterogeneity of keloids and the challenges of targeted therapy [16, 17].

Nintedanib is an oral, small‐molecule, multi‐target TKI that inhibits vascular endothelial growth factor receptors (VEGFRs), fibroblast growth factor receptors (FGFRs), and platelet‐derived growth factor receptors (PDGFRs), pathways implicated in fibroblast activation, angiogenesis, and ECM accumulation [14, 18, 19]. It is approved for idiopathic pulmonary fibrosis and systemic sclerosis‐associated interstitial lung disease, with a well‐characterized safety profile in these approved indications [20, 21, 22]. Preclinical studies have suggested that nintedanib may modulate profibrotic signaling pathways, including TGF‐β/Smad and PI3K/Akt [14]. Nevertheless, whether these pharmacological and preclinical observations translate into clinically meaningful benefit in generalized keloids remains uncertain.

Given the shared fibrotic features between keloids and other fibroproliferative disorders, along with the lack of established systemic options for generalized keloids, we conducted a prospective exploratory pilot study to assess the short‐term safety, tolerability, and exploratory clinical outcomes of oral nintedanib in patients with refractory generalized keloids.

## 2 Materials and Methods

### 2.1 Study Design

This was a single‐center, prospective, non‐randomized, open‐label, assessor‐blinded exploratory pilot study conducted between 2024 and 2025. No placebo or untreated control group was included. The study protocol was approved by the Institutional Ethics Committee of Dermatology Hospital, Southern Medical University (Approval No. 2023068). Written informed consent was obtained from all participants before study initiation. The study comprised a baseline visit and scheduled on‐site visits at Weeks 2, 4, 8 (end of treatment), and 12 (follow‐up), during which exploratory clinical outcome and safety assessments were performed.

### 2.2 Participants

Eligible participants were adults aged 18–65 years with generalized keloids, defined as involvement of three or more distinct anatomical sites with a total lesion count exceeding ten. Key inclusion criteria also included refractory disease, defined as failure to respond to at least two prior local therapies, including intralesional corticosteroids, laser therapy, or surgical excision. Key exclusion criteria included use of systemic corticosteroids or immunosuppressive agents within 8 weeks before baseline; clinically significant hepatic or renal dysfunction, defined as aspartate aminotransferase (AST) or alanine aminotransferase (ALT) > 3 times the upper limit of normal; pregnancy or lactation; prior exposure to systemic TKIs; known hypersensitivity to nintedanib or any of its excipients; and active infections or other severe systemic diseases that could interfere with study assessments.

### 2.3 Dose Allocation and Blinding

Participants were assigned in a 1:1 ratio to receive either low‐dose oral nintedanib, 150 mg once daily, or high‐dose oral nintedanib, 150 mg twice daily, using sealed envelopes prepared before enrollment. Given the small sample size and exploratory pilot design, this allocation procedure was used to balance dose assignment and reduce overt allocation bias, rather than to support confirmatory between‐dose comparisons. Treatment administration was open‐label. Investigators responsible for outcome assessment were blinded to dose allocation, and participants were instructed not to disclose their dosing regimen during assessments in order to reduce assessor bias.

### 2.4 Interventions

All participants received oral nintedanib for 8 consecutive weeks. Protocol‐defined dose modifications or temporary interruptions were permitted for the management of treatment‐emergent adverse events. Concomitant intralesional or systemic therapies for keloids were prohibited throughout the study period. Treatment adherence was monitored by pill counts and review of patient‐maintained medication diaries.

### 2.5 Outcome Evaluation

The primary exploratory clinical outcome was the within‐patient change in the VSS score from baseline to Week 8. The target‐lesion assessment strategy was conceptually informed by established oncology response‐assessment frameworks, such as RECIST 1.1, in which a limited number of prespecified measurable target lesions are followed longitudinally to improve consistency and reproducibility [23, 24]. At baseline, five target lesions per participant were prespecified before treatment initiation and remained unchanged throughout follow‐up.

Target lesions were selected according to predefined and consistently applied criteria. Eligible lesions were required to be clinically measurable, clearly identifiable on standardized photographs, suitable for repeated assessment, and clinically relevant with respect to lesion size, activity, or symptom burden. Where feasible, selected lesions were distributed across different anatomical sites. Lesions with marked ulceration, recent trauma, infection, or difficulty in repeated assessment were avoided.

This target‐lesion approach was intended to support standardized longitudinal assessment of the same lesions across visits, rather than to provide a comprehensive measure of whole‐body disease burden. Target lesions were evaluated using standardized clinical photography and anatomical mapping to ensure evaluation of the same lesions at baseline and at Weeks 2, 4, 8, and 12. The VSS assesses scar vascularity, pigmentation, height, and pliability, with a total score ranging from 0, indicating normal skin, to 15, indicating severe scarring.

Secondary exploratory outcomes included pruritus and pain severity assessed using visual analogue scale (VAS) scores, comprehensive scar assessment using the patient and observer scar assessment scale (POSAS), and health‐related quality of life measured by the dermatology life quality index (DLQI) at each visit.

### 2.6 Clinical, Imaging, and Histological Assessments

Standardized clinical photographs were taken at each visit using a digital camera with fixed lighting, distance, and angle. Standardized ANTERA 3D imaging was performed at baseline and Week 8 to visually document changes in scar vascularity and surface appearance. Punch biopsies from selected lesions were obtained at baseline and Week 8. Hematoxylin and eosin (H&E) staining was used to evaluate general tissue architecture, and Masson's trichrome staining was used to assess collagen fiber density and organization. Histological images were analyzed by two independent pathologists who were blinded to treatment group and time point.

### 2.7 Safety Assessment

Adverse events were recorded at each visit, including type, onset time, duration, severity, and relationship to study drug. TRAEs were defined as AEs with a reasonable temporal relationship to nintedanib administration. Severity of AEs was graded according to the Common Terminology Criteria for Adverse Events (CTCAE), version 5.0. Safety monitoring included serial assessments of vital signs, including blood pressure, heart rate, body temperature; liver function, including AST, ALT, and bilirubin; renal function, including creatinine and urea nitrogen; and complete blood counts, including hemoglobin, white blood cell count, and platelet count, at baseline and Weeks 4, 8, and 12.

### 2.8 Statistical Analysis

Given the small sample size and exploratory pilot design, all clinical outcome analyses were considered descriptive and hypothesis‐generating rather than confirmatory. No formal sample‐size calculation was performed; the sample size was based on feasibility. Baseline characteristics were summarized descriptively and were not formally compared between dose groups. Continuous variables are presented as mean ± standard deviation (SD) and/or median with interquartile range (IQR), as appropriate, and categorical variables are presented as counts and percentages.

For key exploratory clinical outcomes, within‐group changes from baseline to Week 8 were assessed using two‐tailed exact Wilcoxon signed‐rank tests. Exploratory between‐dose comparisons were performed on individual change scores, defined as Week 8 minus baseline, using two‐tailed exact Mann–Whitney U tests. Effect estimates were expressed descriptively as within‐group change from baseline and between‐dose difference in change, each accompanied by 95% confidence intervals where appropriate. Longitudinal outcomes across all study visits were summarized descriptively. No adjustment for multiple comparisons was performed. Accordingly, *p* values were considered nominal and hypothesis‐generating and were not used to support confirmatory efficacy inference. All analyses were based on complete‐case data.

## 3 Results

### 3.1 Participant Characteristics

**FIGURE 1:**
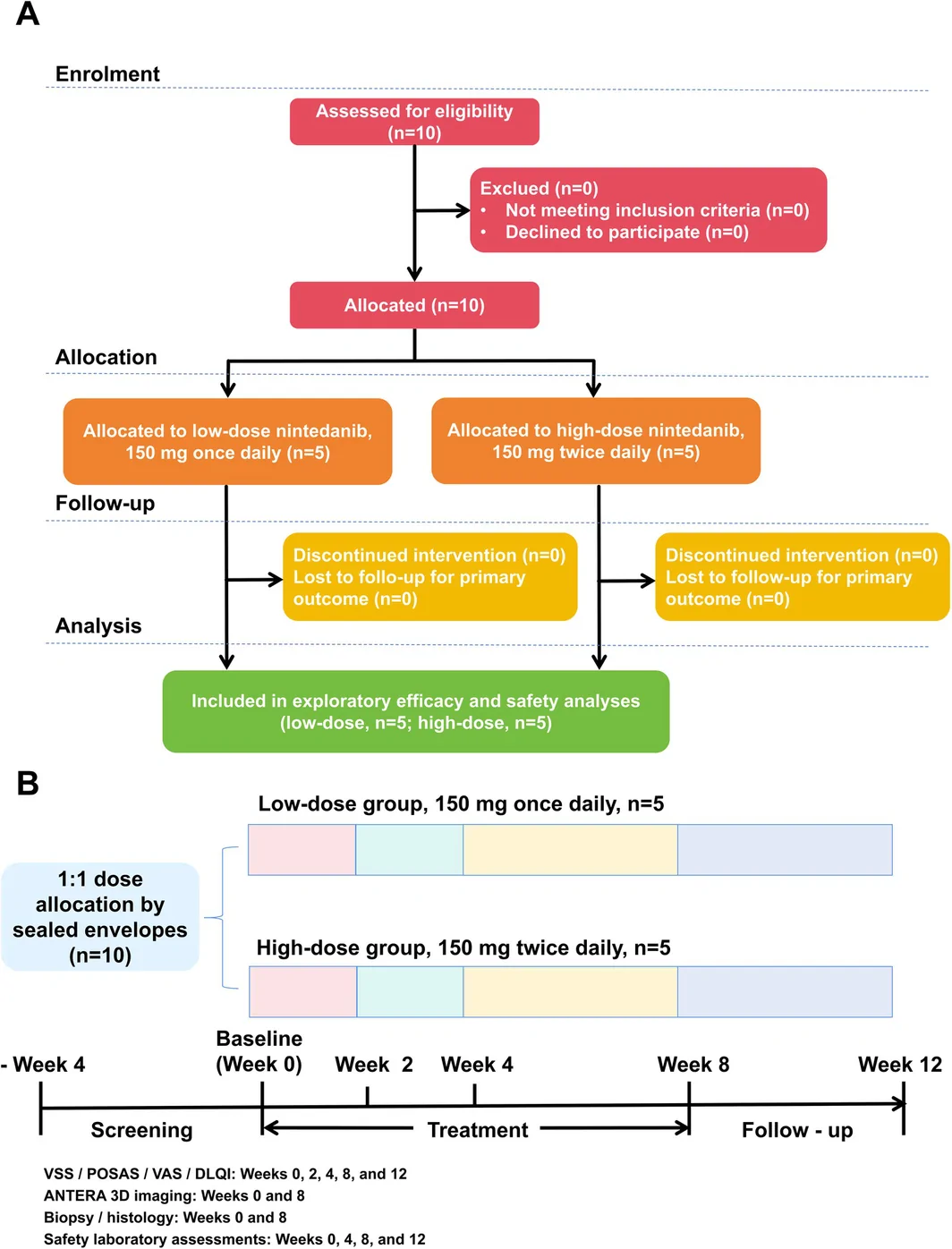
Participant flow and study schedule. (A) Flow chart of participant enrollment, dose allocation, treatment completion, follow‐up, and inclusion in the exploratory analyses. (B) Study schedule, including dose allocation, treatment period, follow‐up period, and assessment time points.

**FIGURE 2:**
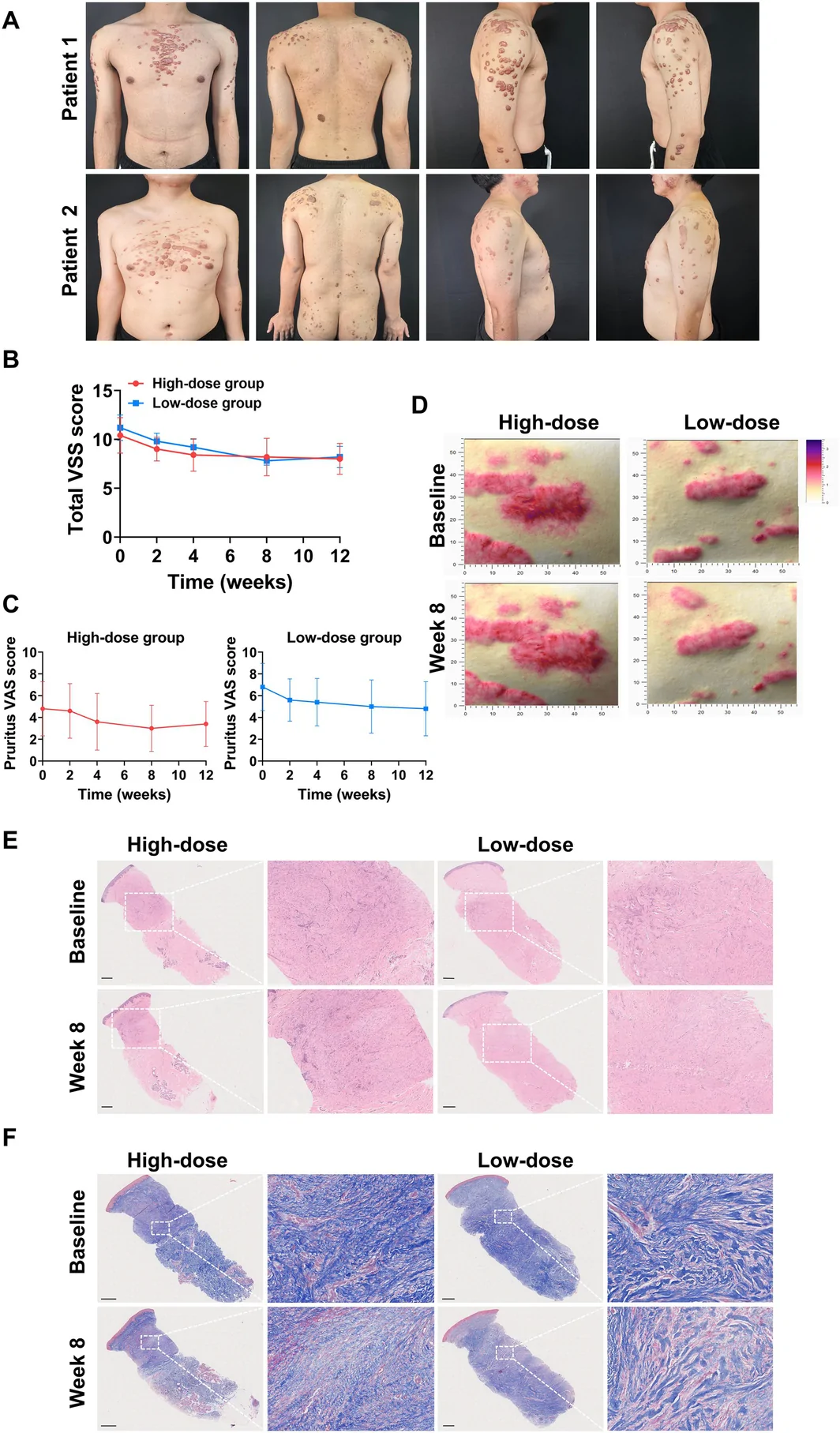
Clinical, imaging, and histological findings during oral nintedanib treatment in generalized keloids. (A) Representative images of generalized keloids. (B) Longitudinal changes in total VSS scores in the high‐dose and low‐dose groups. Values are shown as mean ± SD. Week 12 represents exploratory follow‐up after completion of the 8‐week treatment period. (C) Longitudinal changes in pruritus VAS scores in the high‐dose and low‐dose groups. Values are shown as mean ± SD. Week 12 represents exploratory follow‐up after completion of the 8‐week treatment period. (D) Representative ANTERA 3D images suggesting changes in visible vascularity at baseline and Week 8. (E) H&E staining of keloid tissue at baseline and Week 8 (scale bar = 500 μm). (F) Masson's trichrome staining of keloid tissue at baseline and Week 8 (scale bar = 500 μm).

All 10 participants completed the 8‐week treatment period and follow‐up to Week 12 (Figure 1A). Five participants were assigned to the low‐dose group and five to the high‐dose group (Figure 1B). Given the small exploratory sample size, baseline demographics and clinical characteristics are summarized descriptively in Table 1. Representative clinical images showed extensive keloid involvement across multiple anatomical sites (e.g., chest, back, and shoulders) in participants (Figure 2A).

**TABLE 1:** Baseline participant characteristics.

Characteristic	Low‐dose group (*n* = 5)	High‐dose group (*n* = 5)
Age, years	22.2 ± 2.6	23.8 ± 5.9
male sex, *n* (%)	5 (100)	4 (80)
BMI, kg/m^2^	21.9 ± 4.6	25.2 ± 2.0
Disease duration, years, median (IQR)	6.0 (5.0–8.5)	5.0 (4.0–11.5)
Baseline total VSS score	11.2 ± 1.2	10.4 ± 1.6
Baseline VAS score	2.0 ± 1.6	1.4 ± 2.1

*Note:* Values are mean ± SD.

Abbreviations: BMI, body mass index; IQR, interquartile range; VAS, visual analog scale; VSS, Vancouver scar scale.

### 3.2 Clinical Outcomes

Mean VSS scores decreased from baseline to Week 8 in both groups (Figure 2B). In the low‐dose group, the mean VSS score changed from 11.2 ± 1.3 to 8.2 ± 1.1, corresponding to a mean within‐patient change of −3.0 (95% CI, −5.48 to −0.52; exact Wilcoxon *p* = 0.1250). In the high‐dose group, the mean VSS score changed from 10.4 ± 1.8 to 8.2 ± 1.9, corresponding to a mean within‐patient change of −2.2 (95% CI, −3.82 to −0.58; exact Wilcoxon *p* = 0.0625). The exploratory between‐dose difference in change was 0.8 (95% CI, −1.73 to 3.33; two‐tailed exact Mann–Whitney *p* = 0.3968).

Pruritus VAS scores also decreased over the treatment period (Figure 2C), with mean within‐patient changes of −1.8 (95% CI, −4.76 to 1.16; exact Wilcoxon *p* = 0.2500) in the low‐dose group and −1.8 (95% CI, −3.42 to −0.18; exact Wilcoxon *p* = 0.1250) in the high‐dose group. The exploratory between‐dose difference in change was 0.0 (95% CI, −2.95 to 2.95; two‐tailed exact Mann–Whitney *p* = 0.9841).

Pain VAS scores showed smaller numerical declines, with mean within‐patient changes of −0.6 (95% CI, −1.28 to 0.08; exact Wilcoxon *p* = 0.2500) and −0.8 (95% CI, −2.42 to 0.82; exact Wilcoxon *p* = 0.5000), respectively. The exploratory between‐dose difference in change was −0.2 (95% CI, −1.79 to 1.39; two‐tailed exact Mann–Whitney *p* > 0.9999).

**FIGURE 3:**
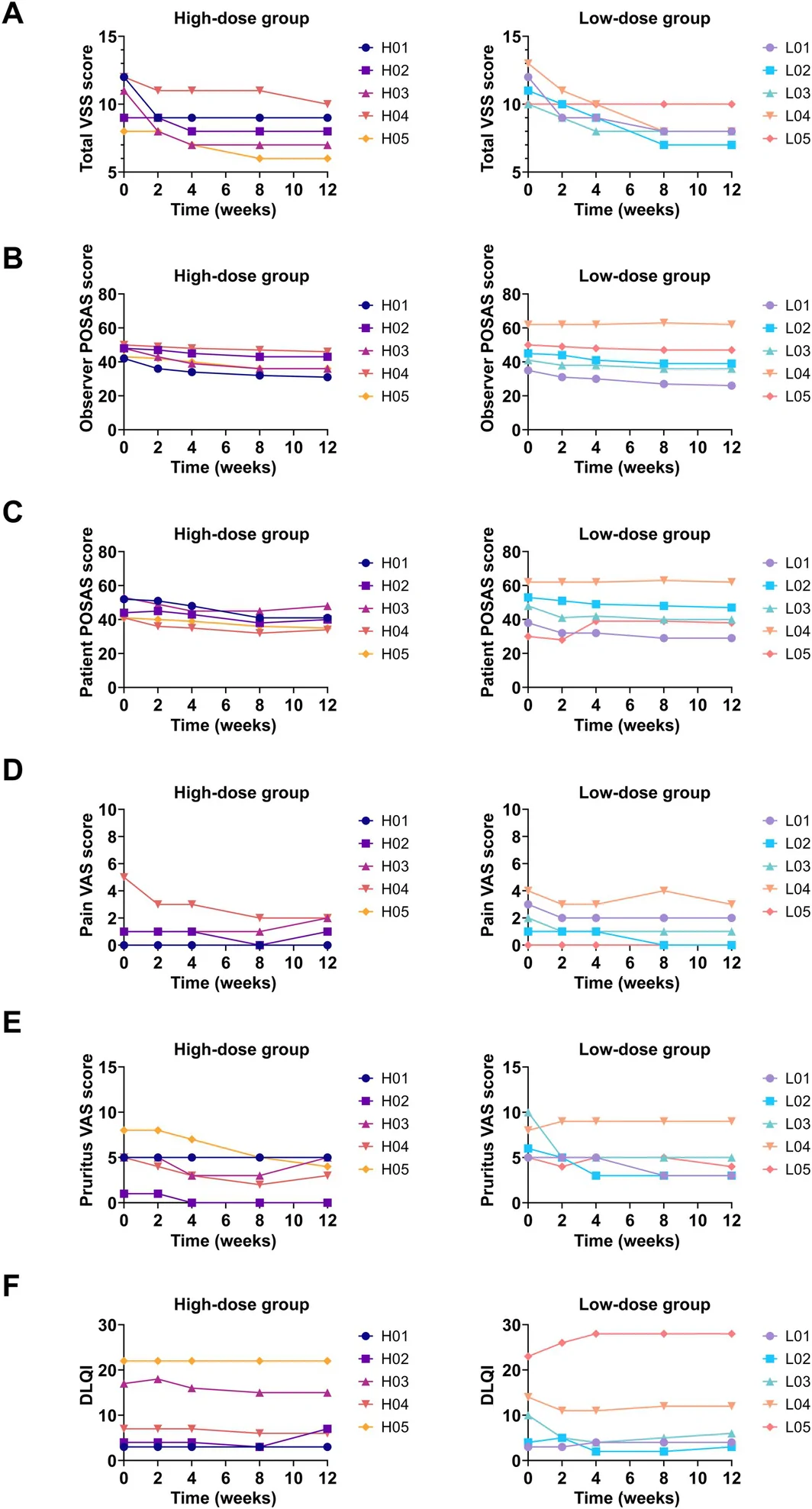
Individual participant trajectories for exploratory clinical and patient‐reported outcomes. (A) Total VSS scores. (B) Observer POSAS scores. (C) Patient POSAS scores. (D) Pain VAS scores. (E) Pruritus VAS scores. (F) DLQI scores. H01–H05 denote participants in the high‐dose group, and L01–L05 denote participants in the low‐dose group. Week 12 represents the exploratory follow‐up assessment after completion of the 8‐week treatment period.

Representative ANTERA 3D images suggested reduced visible vascularity at Week 8 in selected participants (Figure 2D). Histological evaluation showed descriptive changes at Week 8 compared with baseline: H&E staining suggested reduced cellularity, predominantly involving fibroblasts and inflammatory cells, and Masson's trichrome staining suggested decreased collagen fiber density and more organized fiber alignment (Figure 2E,F). By contrast, observer‐ and patient‐reported POSAS scores and DLQI showed limited change over the 8‐week treatment period (Figure 3).

At the exploratory Week 12 follow‐up, the patterns observed for VSS scores, pruritus VAS scores, and visible vascularity were maintained descriptively in some participants, although no formal statistical analysis was performed for this follow‐up time point.

### 3.3 Safety and Tolerability

In this small exploratory cohort, short‐term oral nintedanib was generally tolerated over the 8‐week treatment period. Five participants (50%) experienced at least one TRAE, most commonly diarrhea, elevated AST, and elevated ALT (Table 2). Specifically, diarrhea occurred in 2 (40%) participants in the low‐dose group and 3 (60%) in the high‐dose group, all of which were Grade 1. Elevated ALT was observed in 1 (20%) Grade 1 case in the low‐dose group and 3 (60%) cases (2 Grade 1, 1 Grade 2) in the high‐dose group. Elevated AST occurred in 2 (40%) Grade 1 cases in the high‐dose group, with no cases in the low‐dose group. All TRAEs were reversible: diarrhea was managed with symptomatic treatment (oral rehydration), and elevated transaminases resolved spontaneously without dose modification or treatment interruption. No Grade ≥ 3 AEs, serious AEs, or treatment discontinuations occurred during the study.

**TABLE 2:** Treatment‐related adverse events.

Adverse Event	Low‐dose group (*n* = 5), *n* (%)	High‐dose group (*n* = 5), *n* (%)	Total (*n* = 10), *n* (%)	CTCAE Grade 1, *n*	CTCAE Grade 2, *n*
Diarrhea	2 (40)	3 (60)	5 (50)	5	0
ALT elevation	1 (20)	3 (60)	4 (40)	3	1
AST elevation	0 (0)	2 (40)	2 (20)	2	0

Abbreviations: ALT, alanine aminotransferase; AST, aspartate aminotransferase; CTCAE, common terminology criteria for adverse events.

## 4 Discussion

Generalized keloids represent a severe and therapeutically challenging manifestation of pathological scarring, with extensive lesion burden, chronic symptoms, and substantial psychosocial impact, yet lacking established systemic treatment options [9]. In this prospective exploratory pilot study, 8‐week oral nintedanib was associated with early favorable changes in selected clinician‐rated and symptom‐based measures, including VSS, pruritus, and visible vascularity. However, given the small sample size, uncontrolled design, and short follow‐up, these findings should be interpreted as preliminary observations rather than evidence of treatment efficacy.

The observed changes were not consistent across all outcome domains. Although VSS and pruritus showed early favorable trends, patient‐reported outcomes, including POSAS and DLQI, changed little over the 8‐week treatment period. This discrepancy is clinically important and suggests that short‐term changes in selected clinician‐rated or symptom‐based measures may not translate into meaningful patient‐perceived improvement. In addition, the 8‐week treatment duration and Week 12 follow‐up are insufficient to assess durable scar remodeling, long‐term symptom control, quality‐of‐life benefit, or recurrence. Therefore, the present study is best viewed as an exploratory assessment of short‐term clinical and histological observations rather than as a demonstration of sustained antifibrotic efficacy.

The observed change in visible vascularity is biologically plausible in view of the known pharmacological profile of nintedanib, which targets VEGFR, FGFR, and PDGFR signaling pathways implicated in angiogenesis, fibroblast activation, and extracellular matrix remodeling. However, no biomarker, molecular, or pathway analyses were performed in this study. Accordingly, the present data cannot determine whether the observed clinical or histological findings were mediated through these pathways. The pharmacological rationale for nintedanib should therefore be regarded as hypothesis‐generating and as a basis for future translational studies. Previous clinical studies of targeted therapies for keloids have mostly involved localized disease and small cohorts, and evidence for systemic treatment of generalized keloids remains limited [16, 17]. In this context, the present study provides preliminary prospective clinical observations in a difficult‐to‐treat generalized keloid population. Nevertheless, controlled studies with larger sample sizes, validated global disease‐burden assessments, longer treatment duration, and integrated biomarker analyses are required to determine whether oral nintedanib has clinically meaningful benefit in this setting.

The safety findings should also be interpreted cautiously. No serious adverse events, Grade ≥ 3 adverse events, or treatment discontinuations occurred during the 8‐week treatment period. The most common treatment‐related adverse events were mild diarrhea and reversible transaminase elevation. These findings suggest generally manageable short‐term tolerability in this small cohort, but they do not establish the safety profile of nintedanib in generalized keloid populations, particularly with longer exposure. The higher frequency of liver enzyme abnormalities in the high‐dose group suggests the need for close laboratory monitoring in future studies.

### 4.1 Limitations

This study has several important limitations. First, the absence of a placebo or untreated control group represents a major limitation and precludes causal attribution of the observed changes to nintedanib. Natural disease fluctuation, regression to the mean, scoring variability, expectation effects, and the nonspecific effects of structured follow‐up may have contributed to the observed findings. Second, the sample size was very small and no formal sample‐size calculation was performed, limiting statistical power, precision of effect estimates, and the reliability of between‐dose comparisons. Accordingly, all clinical outcome analyses should be regarded as exploratory and hypothesis‐generating rather than confirmatory. Third, the open‐label design may have increased susceptibility to expectation bias, although outcome assessors were blinded to dose allocation. Fourth, the target‐lesion approach, while conceptually informed by established oncology response‐assessment frameworks and useful for standardized longitudinal assessment, has not been validated for generalized keloids. Assessment of five prespecified target lesions may not fully capture the total lesion burden, anatomical distribution, or morphological heterogeneity, and lesion‐selection bias could not be excluded. Future studies should incorporate validated global disease‐burden measures, such as whole‐body lesion mapping, total lesion counts, lesion‐area or volumetric assessment, standardized global photography, and patient‐centered outcome measures. Fifth, the 8‐week treatment period and short post‐treatment follow‐up were insufficient to assess durable scar remodeling, long‐term symptom control, quality‐of‐life benefit, recurrence, or delayed safety events. Sixth, no biomarker, molecular, or pathway analyses were performed, limiting mechanistic interpretation of the clinical, imaging, and histological observations. Finally, the predominance of male participants and the single‐center design may limit the generalizability of the findings.

## 5 Conclusion

In this prospective exploratory pilot study of refractory generalized keloids, oral nintedanib was associated with short‐term favorable changes in selected clinician‐rated and symptom‐based measures, with limited changes in patient‐reported outcomes. Treatment was generally tolerated over 8 weeks. Given the uncontrolled design, small sample size, and short follow‐up, these findings remain preliminary and warrant confirmation in adequately powered controlled studies.

## Author Contributions

Chenmei Liu, Lian Zhang, and Bin Yang contributed substantially to study conception and design; Dan Sun, Aiqing Lin, and Renliang He acquired the data; Chenmei Liu drafted the manuscript, and Lian Zhang revised its key intellectual content; Renliang He, and Bin Yang gave final approval to the version for publication.

## Funding

This work was supported by the National Natural Science Foundation of China 82303064.

## Ethics Statement

Approved by the Ethics Committee of Dermatology Hospital of Southern Medical University (Approval No. 2023068). Registered at ChiCTR2400089943.

## Consent

Written informed consent obtained.

## Conflicts of Interest

The authors declare no conflicts of interest.

## Data Availability

The data that support the findings of this study are available from the corresponding author upon reasonable request.
